# Phosphatidylinositol-3-Kinase (PI3K) and Histone Deacetylase (HDAC) Multitarget Inhibitors: An Update on Clinical and Preclinical Candidates

**DOI:** 10.3390/ph19010130

**Published:** 2026-01-12

**Authors:** Alef D. S. Lima, Lídia M. Lima

**Affiliations:** 1 Laboratory for the Evaluation and Synthesis of Bioactive Substances (LASSBio^®^), National Institute of Science and Technology for Drugs and Medicines (INCT-INOFAR), Federal University of Rio de Janeiro, Rio de Janeiro 21941-902, RJ, Brazil; aleflimaeq@gmail.com; 2 Graduate Program in Chemistry, Institute of Chemistry, Federal University of Rio de Janeiro, Rio de Janeiro 21941-909, RJ, Brazil

**Keywords:** cancer, multitarget-directed ligands, HDAC and PI3K inhibition

## Abstract

Phosphatidylinositol-3-kinases (PI3Ks) constitute an important validated therapeutic class involved in crucial cellular processes, and their dysregulation is associated with cancer initiation and progression. Nonetheless, intrinsic and acquired resistance mechanisms associated with PI3K pathway modulation have underscored the need for alternative therapeutic strategies. In this context, recent studies have shown that simultaneous inhibition of PI3K and histone deacetylases (HDAC) promotes synergistic antitumor effects in different cancer cell lines. HDACs are validated epigenetic targets that are extensively explored in clinical practice and have a pharmacophore with versatility for structural modifications, which facilitates the design of multitarget inhibitors. This review examines the rational design and synthetic evolution of dual PI3K/HDAC inhibitors, an area catalyzed by the development of fimepinostat, the first clinically evaluated agent exhibiting potent and balanced inhibition of both targets. We provide a critical overview of PI3K/HDAC multitarget inhibitors reported in recent years that have progressed to preclinical or clinical investigation, discussing the structural frameworks employed, medicinal chemistry strategies adopted, and structure–activity relationships established. Particular attention is given to advantageous molecular features as well as challenges related to toxicity, pharmacokinetic behavior, and pharmacodynamic modulation. From this comprehensive analysis, we outline key considerations and emerging design principles that may inform the next generation of PI3K/HDAC multitarget drug candidates. Insights derived from the diversity of chemical scaffolds, activity profiles, and selectivity patterns described herein may support the development of innovative therapeutic agents capable of overcoming current limitations in anticancer treatment.

## 1. Introduction

Throughout the history of drug discovery, the field has been largely shaped by the classical “one molecule–one target–one disease” paradigm, which emphasizes the design of highly selective ligands to modulate individual biological targets implicated in pathological conditions [1]. However, technological advances across the biological, chemical, and health sciences have increasingly revealed the multifactorial and interconnected nature of chronic non-communicable diseases, thereby opening the way for the intentional design of multitarget therapeutic agents [2,3]. This conceptual shift has been particularly impactful in oncology, where tumor initiation and progression frequently involve the simultaneous dysregulation of multiple receptors and signaling pathways [4].

Pharmacological cancer therapy rarely relies on monotherapy. Instead, treatment commonly employs combinations of single-agent drugs—often referred to as drug combination therapy or antitumor “cocktails”—in which multiple agents are administered concomitantly, each acting through distinct yet complementary mechanisms of action [5]. Combination regimens may take the form of co-administered agents, fixed-dose combinations (single formulations containing multiple active pharmaceutical ingredients), or multitarget-directed ligands (MTDLs). The latter correspond to a single molecular entity capable of modulating more than one biological target simultaneously. Introduced by Morphy and Rankovic in 2005, the MTDL concept has since emerged as a compelling alternative to Paul Ehrlich’s early-20th-century “magic bullet” paradigm [6].

The MTDL strategy involves the use of a single active compound able to interact with two or more targets either vertically—modulating proteins within the same signaling pathway—or horizontally—acting on targets distributed across distinct biological pathways. Such simultaneous modulation can produce synergistic therapeutic effects, thereby offering a powerful approach to address complex diseases like cancer [7]. Unlike traditional combination therapies, MTDLs circumvent several limitations, including poor patient adherence, disparities in pharmacokinetic or pharmacodynamic profiles between co-administered drugs, and the risk of drug–drug interactions. Moreover, multitarget ligands may reduce required therapeutic doses, minimize systemic toxicity, and mitigate the emergence of resistance mechanisms [8].

**Figure 1:**
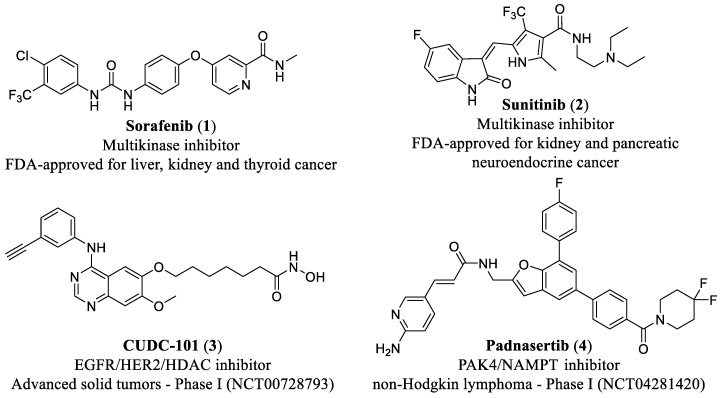
Chemical structures of FDA-approved multitarget inhibitors 1 and 2 and clinical candidates 3 and 4.

Despite the inherent challenges—such as achieving appropriate potency and selectivity across multiple targets within a single scaffold while ensuring adequate bioavailability—numerous MTDLs have achieved regulatory approval or entered clinical development, demonstrating promising therapeutic potential in oncology. Representative examples are depicted in Figure 1 [1,9].

Among the molecular targets currently prioritized in the design of multitarget-directed ligands (MTDLs) for oncological applications, phosphoinositide 3-kinases (PI3Ks) and histone deacetylases (HDACs) have emerged as particularly relevant due to their pivotal roles in cellular homeostasis, epigenetic regulation, and tumor progression. In this review, we provide a comprehensive examination of the key signaling pathways governed by these enzyme families, outline the principal structural classes and pharmacological profiles of their inhibitors, and present a detailed medicinal chemistry analysis of dual PI3K/HDAC modulators described to date. Emphasis is placed on molecular design strategies, scaffold-hopping approaches, structure–activity relationships, and the physicochemical and pharmacokinetic considerations that have shaped the development of this multitarget chemotype.

### 1.1. Phosphatidylinositol-3-Kinases (PI3K)

The phosphoinositide 3-kinase (PI3K) signaling pathway constitutes a central intracellular cascade with profound influence on key cellular processes, including growth, motility, proliferation, survival, metabolic regulation, and angiogenesis [10,11]. PI3Ks comprise a family of lipid kinases located predominantly at the plasma membrane, where they catalyze the phosphorylation of the 3′-hydroxyl group of the inositol ring in phosphoinositides using adenosine triphosphate (ATP) as the phosphate donor [12]. Based on structural organization, substrate preference, and regulatory mechanisms, PI3Ks are classified into three major classes—classes I, II, and III. Class I PI3Ks, the most extensively characterized, are subdivided into class IA (isoforms α, β, and δ) and class IB (γ). Notably, class IA isoforms are predominantly implicated in the initiation and maintenance of diverse human malignancies [10,13].

Dysregulation of the PI3K pathway plays a pivotal role in tumorigenesis and cancer progression. Oncogenic activation frequently arises from aberrant PI3K expression, loss or inactivation of the lipid phosphatase PTEN (phosphatase and tensin homolog deleted on chromosome 10), mutations in catalytic subunits (most commonly PIK3CA), or alterations in PI3K-associated oncogenes [14]. As a consequence, the PI3K pathway has emerged as a high-value therapeutic target in oncology, driving the intensive development of small-molecule inhibitors over recent decades.

**Figure 2:**
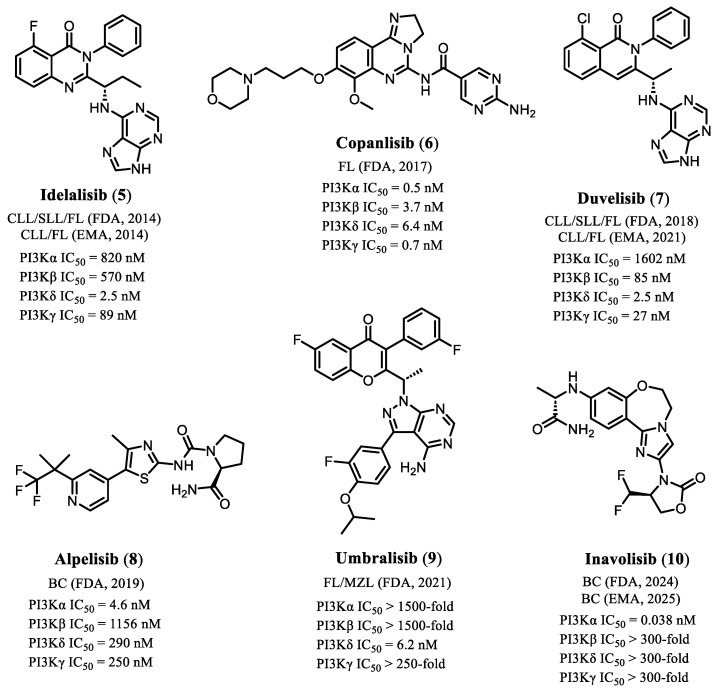
Chemical structures of PI3K inhibitors approved for clinical use. CLL; chronic lymphocytic leukemia, SLL; small lymphocytic leukemia, FL; follicular lymphoma, MZL; marginal zone lymphoma, BC; breast cancer, FDA; U.S. Food and Drug Administration, EMA; European Medicines Agency [15,16,17,18,19,20].

To date, six PI3K inhibitors have received FDA approval for clinical use: Idelalisib (**5**), Copanlisib (**6**), Duvelisib (**7**), Alpelisib (**8**), Umbralisib (**9**), and Inavolisib (**10**) (Figure 2) [15,16,17,18,19,20]. With the exception of Alpelisib (**8**) and the more recently approved Inavolisib (10)—both indicated for the treatment of solid tumors, particularly hormone receptor–positive breast cancer—approved PI3K inhibitors have been primarily directed toward hematologic malignancies. The therapeutic relevance of these agents underscores both the importance and the complexity of selectively modulating this pathway in cancer therapy.

It is important to highlight that several clinical indications for approved PI3K inhibitors were subsequently withdrawn, particularly those granted through the FDA’s accelerated approval pathway, due to the emergence of serious safety concerns or insufficient evidence of clinical benefit in post-marketing confirmatory trials [21]. In 2022, the indications of Idelalisib (**5**) for small lymphocytic lymphoma (SLL) and follicular lymphoma (FL) were voluntarily withdrawn by the manufacturer (Gilead Sciences, Inc.) after post-approval studies demonstrated increased mortality among treated patients [22,23]. Idelalisib currently carries a boxed warning outlining risks such as severe hepatotoxicity, infections, diarrhea/colitis, and pneumonitis.

Similarly, in 2022 the FDA revoked the indication of Duvelisib (**7**) for FL following evidence of increased fatal and/or severe toxicities, including infections and gastrointestinal adverse events, also reflected in its boxed warning. The approvals for Umbralisib (**9**) and Copanlisib (**6**) were withdrawn in 2022 and 2023, respectively, as accumulating data indicated elevated mortality risk associated with their approved uses. Despite these regulatory setbacks, PI3K inhibitors continue to be evaluated in clinical trials for new therapeutic indications, employing modified dosing schedules or novel combination regimens. This continued clinical interest underscores the therapeutic relevance of modulating PI3K signaling in oncology [22,23].

Although promising antitumor activity has been observed in selected malignancies, the efficacy of PI3K inhibitors as monotherapies remains limited, largely due to compensatory activation of parallel or downstream signaling pathways that ultimately promote treatment resistance [24]. This challenge has stimulated growing interest in combining PI3K inhibitors with other anticancer agents, as well as in the rational design of multitarget PI3K inhibitors capable of simultaneously modulating complementary molecular pathways [25,26]. Such strategies aim to enhance therapeutic efficacy, suppress adaptive resistance mechanisms, and broaden the clinical potential of PI3K-targeted therapies. In this context, a promising alternative that has been explored is the concomitant inhibition of PI3K and histone deacetylase, since the regulation of histone and non-histone protein acetylation levels modulates cellular events that synergize with the modulation of the PI3K signalling pathway. Studies have shown that PI3K inhibition significantly increases the cytotoxicity of HDAC inhibitors in lung cancer and chronic myeloid leukemia cells, indicating that dual inhibition of these two targets may be very effective [27,28].

### 1.2. Histone Deacetylases (HDAC)

Histone deacetylases (HDACs) represent one of the most extensively investigated classes of epigenetic regulators in oncology. These enzymes exert fundamental roles in transcriptional control and gene expression by modulating chromatin compaction and protein acetylation status, thereby influencing key cellular processes such as proliferation, differentiation, migration, and apoptosis [29,30,31]. The human HDAC family comprises 18 isoforms, grouped into four classes based on sequence homology, domain architecture, and cofactor dependency. Classes I (HDAC1, HDAC2, HDAC3, and HDAC8), IIa (HDAC4, HDAC5, HDAC7, and HDAC9), IIb (HDAC6 and HDAC10), and IV (HDAC11) constitute the zinc-dependent HDACs, whereas class III (sirtuins) are nicotinamide adenine dinucleotide (NAD^+^)-dependent deacylases [32]. HDACs catalyze the removal of acetyl groups from ε-N-lysine residues of histone and non-histone proteins, thereby regulating chromatin accessibility and diverse acetylation-dependent signaling pathways. Overexpression or dysregulation of specific HDAC isoforms has been implicated in the initiation and progression of multiple cancer types, reinforcing their therapeutic relevance as molecular targets for anticancer drug development [33].

**Figure 3:**
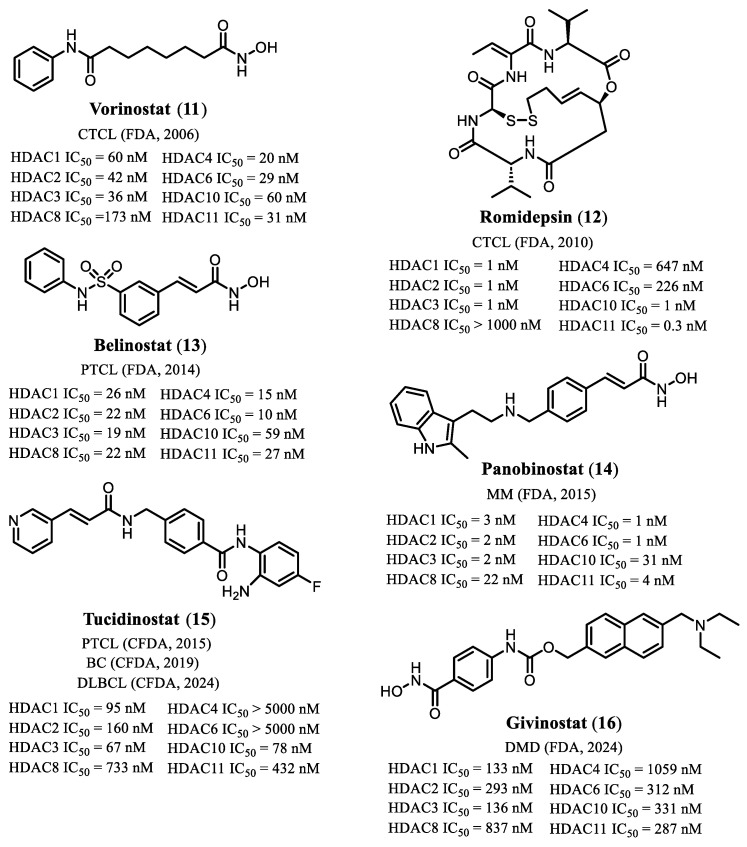
Chemical structures of HDAC inhibitors approved for clinical use. BC, breast cancer; CTCL, cutaneous T-cell lymphoma; DLBCL, diffuse large B-cell lymphoma; DMD, Duchenne muscular dystrophy; MM, multiple myeloma; PTCL, peripheral T-cell lymphoma [34,35,36,37,38,39].

To date, six histone deacetylase inhibitors (HDACis) have been approved for clinical use in humans. Five of these agents—vorinostat (**11**), romidepsin (**12**), belinostat (**13**), panobinostat (**14**), and tucidinostat (**15**)—are indicated for oncological applications, particularly hematological malignancies [34,35,36,37,38]. In 2024, givinostat (**16**, Figure 3) received FDA approval for the treatment of Duchenne muscular dystrophy, becoming the first HDAC inhibitor authorized for a non-oncological indication, thereby expanding the therapeutic landscape of this drug class [39].

Approved HDAC inhibitors (HDACis) continue to be investigated in clinical trials for additional therapeutic indications, and numerous next-generation inhibitors remain under active preclinical and clinical development [40]. Despite their validated clinical efficacy in hematological malignancies, the utility of HDACis as monotherapies is limited, particularly in the context of solid tumors. Several factors contribute to this reduced effectiveness. Solid tumors typically consist of more differentiated cell populations that display diminished sensitivity to epigenetic modulation. Moreover, HDACi monotherapy often triggers compensatory activation of alternative pro-survival and proliferative signaling pathways, including the PI3K pathway, ultimately promoting therapeutic resistance [41,42,43].

These observations reinforce the rationale for combinatorial strategies or for the rational design of multitarget agents capable of concurrently inhibiting both HDACs and PI3Ks. Such dual-modulation approaches may enhance antitumor efficacy, overcome adaptive resistance mechanisms, and broaden the clinical applicability of HDAC-based therapies.

## 2. PI3K and HDAC Signalling Pathway Inhibition

**Figure 4:**
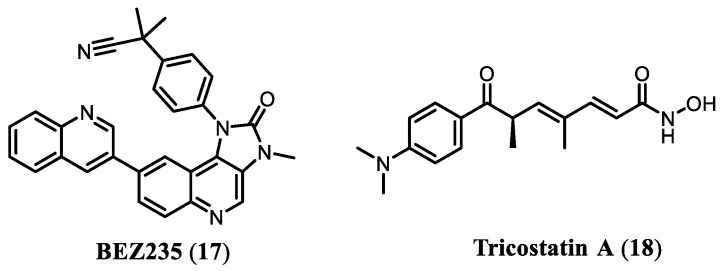
Chemical structures of compounds **17** and **18**.

One of the earliest demonstrations of the therapeutic advantage of concomitant PI3K and HDAC inhibition was reported by Ellis et al., 2013, who described a pronounced synergistic effect arising from the combined administration of panobinostat (**14**, a pan-HDAC inhibitor) and BEZ235 (**17**, a dual PI3K/mTOR inhibitor) (Figure 4) [44]. In in vivo experiments conducted in male SCID mice bearing subcutaneous PC-3 or PC3-AR prostate tumor xenografts, the combination of compounds **14** and **17** produced a significantly greater reduction in tumor volume than either agent administered as monotherapy.

Mechanistic studies revealed that the dual-drug regimen induced heightened intratumoral DNA damage while concurrently decreasing DNA repair capacity. Furthermore, the combination led to a marked reduction in phosphorylated protein kinase B (p-AKT) levels—consistent with PI3K pathway suppression—thereby impairing a critical survival signaling axis.

Collectively, these findings demonstrated in animal models that dual PI3K/HDAC pathway blockade can produce robust synergistic antitumor activity and provided strong preclinical evidence supporting the development of combination-based therapeutic strategies for the treatment of prostate cancer in animal models [44].

In a related study, Piao et al. (2016) evaluated the therapeutic potential of combining BEZ235 (**17**) with trichostatin A (**18**), a prototypical pan-HDAC inhibitor, for the treatment of lung cancer (Figure 4) [26]. The authors demonstrated that the combination exerted a marked synergistic effect in non-small-cell lung cancer (NSCLC) cell lines (A549 and H460), resulting in enhanced inhibition of cell proliferation, induction of apoptosis, and suppression of tumor growth and metastatic dissemination in vivo [26].

Building upon these findings, the development of multitarget HDAC/PI3K inhibitors has gained substantial prominence in both the scientific and patent literature, with several candidates currently advancing through preclinical and clinical evaluation. To provide a comprehensive and updated overview of the state of the art in dual HDAC–PI3K modulation, we conducted a systematic search in the Scopus and Cortellis databases, as well as in clinical trial registries maintained by regulatory agencies. The search employed the keywords “HDAC” and “PI3K Dual Inhibitor” and covered the period from 2015 to 2025.

This strategy identified 72 scientific articles and 81 patents. From these, nine entries were selected based on the criteria of (i) reporting compounds with confirmed multitarget mechanisms involving both HDAC and PI3K inhibition, and (ii) having documented progression into preclinical or clinical development. These inhibitors are summarized and discussed in detail in the following sections.

## 3. Multitarget PI3K/HDAC Inhibitors in Clinical Phase

**Figure 5:**
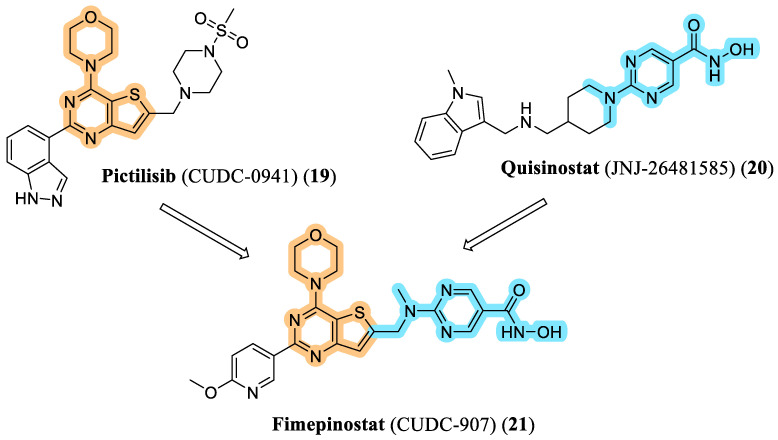
Rational design of fimepinostat (21).

The first PI3K/HDAC multitarget inhibitor to advance into clinical evaluation was fimepinostat (CUDC-907, **21**). Developed by Curis, this morpholinopyrimidine derivative represents the earliest dual HDAC–PI3K inhibitor to receive FDA Fast Track designation (2018) for the treatment of relapsed or refractory diffuse large B-cell lymphoma (DLBCL) [45]. The design of CUDC-907 (**21**) was guided by a molecular hybridization strategy that combined key pharmacophoric elements of quisinostat (JNJ-26481585, **20**), a potent pan-HDAC inhibitor, and pictilisib (CUDC-0941, **19**), a pan-PI3K inhibitor (Figure 5) [46].

**Figure 6:**
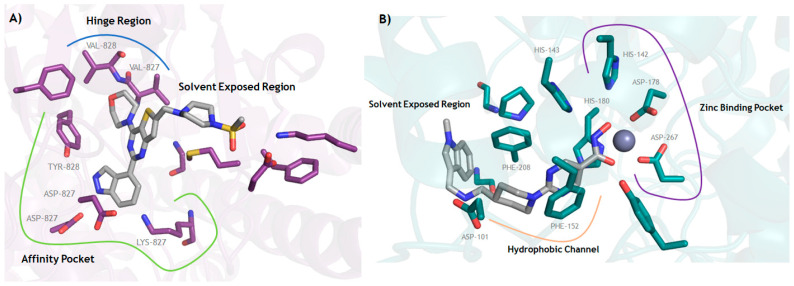
Interaction modes of (**A**) pictilisib (**19**) with PI3Kδ (PDB: 2WXP) and (**B**) quisinostat (**20**) with HDAC8 (PDB: 6HSK). Images generated using PyMOL (version 1.5.0.5) software.

The hydroxamic acid moiety—an essential zinc-binding group (ZBG) responsible for chelating the catalytic Zn^2+^ ion within the HDAC active site—was retained, along with the pyrimidine linker that properly positions the molecule within the HDAC catalytic channel, as supported by co-crystal structural analyses (Figure 6). Within the solvent-exposed region of the HDAC binding pocket, structural space permitted incorporation of the morpholinopyrimidine fragment, which functions as the hinge-binding pharmacophore necessary for engagement with the PI3K catalytic domain. Additionally, the 2-methoxypyridine group was added to enable complementary interactions at the kinase affinity pocket, contributing to high-affinity PI3K inhibition (Figure 6) [47,48,49].

Table 1 summarizes the biochemical potency data for fimepinostat (CUDC-90, **21**) and its precursor prototypes, revealing IC_50_ values in the low-nanomolar range across both HDAC and PI3K targets [46,47,48]. Compound **21** exhibited robust antiproliferative activity in a broad panel of cancer cell lines, including those derived from solid tumors and hematologic malignancies, consistently outperforming the effects of isolated HDAC or PI3K inhibition as well as their combination as separate agents.

Mechanistically, treatment with CUDC-907 induced marked increases in acetylated tubulin, acetylated histone H3, p53, and acetylated p21—biochemical hallmarks of effective HDAC inhibition—alongside a significant reduction in phosphorylated AKT levels, confirming suppression of PI3K/AKT pathway signaling [46,50]. These dual pharmacodynamic signatures support the proposed multitarget mechanism of action and underscore the enhanced biological efficacy afforded by simultaneous HDAC and PI3K modulation.

Fimepinostat (**21**) remains under active clinical investigation for both solid tumors and hematologic malignancies, as well as for emerging non-oncological indications (Table 2). Notably, results from a phase I clinical trial (NCT01742988) in patients with lymphoma demonstrated that CUDC-907 (**21**) was well tolerated, with no dose-limiting toxicities or serious adverse events reported at the evaluated dose levels. Based on these findings, the FDA granted orphan drug designation to fimepinostat in 2018 for the treatment of relapsed or refractory diffuse large B-cell lymphoma (DLBCL) and for nuclear protein in testis (NUT) midline carcinoma [51,52]. These regulatory designations underscore the therapeutic promise of dual HDAC–PI3K inhibition and support continued clinical development across diverse disease settings.

**Figure 7:**
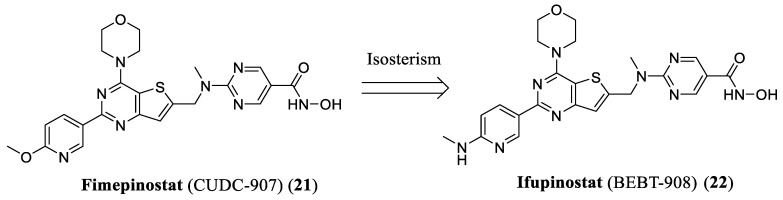
Rational design of ifupinostat (**22**).

Ifupinostat (BEBT-908, 22) is a structural analog of fimepinostat (**21**) originally discovered by Curis and subsequently licensed to Guangzhou BeBetter Medicine Technology Company. In July 2025, it received conditional approval from the China Food and Drug Administration (CFDA) for the treatment of adult patients with relapsed or refractory diffuse large B-cell lymphoma (R/R DLBCL) who had failed at least two prior systemic therapies. With this authorization, ifupinostat became the first dual HDAC/PI3K inhibitor to obtain marketing approval in any country (Figure 7) [53].

Although detailed enzymatic potency data for ifupinostat (**22**) against HDAC and PI3K isoforms have not yet been disclosed, the company’s regulatory communication indicates that the compound exhibits a dual inhibitory mechanism targeting HDACs and PI3Kα, and has shown substantial antitumor activity in lymphoma models [54]. In a phase IIb clinical trial (NCT06074107) supporting its conditional approval, ifupinostat achieved an overall response rate (ORR) of 54.6% and a median overall survival (OS) of 8.8 months—nearly double that observed with standard first-line therapies.

Safety outcomes reported in the same study demonstrated that most hematologic adverse events were reversible and occurred without major complications such as severe bleeding or treatment-related mortality. Furthermore, non-hematologic toxicities were less frequent and generally milder compared with those associated with existing approved therapies for this indication. Ifupinostat (**22**) remains under active clinical investigation for R/R DLBCL, including combination regimens with additional chemotherapeutic agents (Table 2) [52], further supporting its potential as a next-generation multitarget therapeutic agent.

## 4. Multitarget PI3K/HDAC Inhibitors in Pre-Clinical Phase

Based on the literature search conducted for this review, eight dual HDAC/PI3K inhibitors currently supported by preclinical data were identified. These compounds will be discussed in the following sections according to the nature of their zinc-binding group (ZBG), a key structural determinant of HDAC inhibitory activity. Accordingly, the identified molecules are categorized into two major classes: hydroxamic acids and *o*-aminobenzamides.

### 4.1. Multitarget PI3K/HDAC Inhibitors Bearing Hydroxamic Acid Group

**Figure 8:**
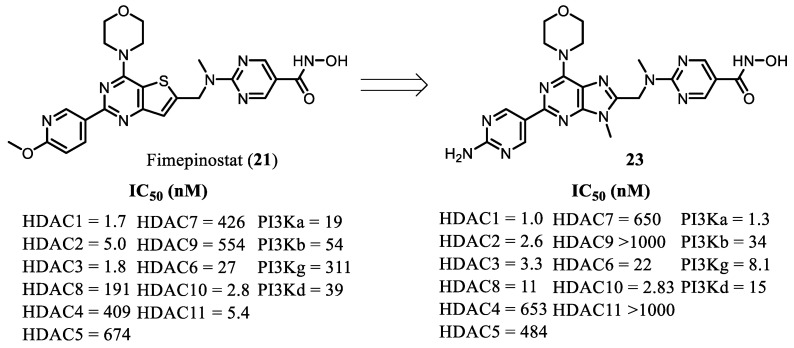
Enzymatic activities for HDAC and PI3K of fimepinostat (**21**) and compound **23** [55].

Inspired by the promising therapeutic profile of fimepinostat (**21**), Chen et al., 2016 [55], designed a series of structural analogues incorporating the morpholinopurine scaffold. This medicinal chemistry optimization effort led to the identification of compound **23**, a dual HDAC/PI3K inhibitor that exhibited enhanced potency against both targets compared with the parent compound (Figure 8).

In vitro studies demonstrated that compound **23** effectively inhibited the phosphorylation of key components of the PI3K signaling axis, including PI3K, mTOR, and AKT, while simultaneously increasing acetylation levels of histone H3 and α-tubulin—pharmacodynamic hallmarks consistent with HDAC inhibition. These findings confirm the multitarget inhibitory profile of this molecule. However, when evaluated in vivo using the MV4-11 xenograft model in NOD/SCID mice, compound **23** exhibited the weakest antiproliferative activity within its series and displayed poor aqueous solubility coupled with toxicity concerns. These limitations indicate that further structural optimization is warranted to enhance solubility and mitigate potential toxicophoric liabilities [55].

**Figure 9:**
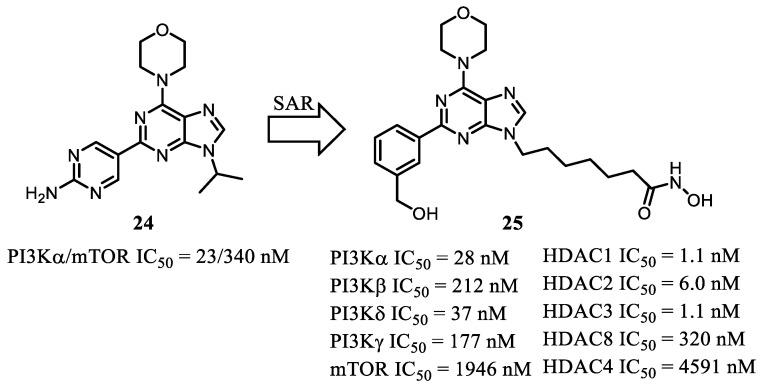
Identification of compound **25** [56].

In a subsequent study, Chen et al. (2018) [56] designed and synthesized PI3K/HDAC multitarget inhibitors incorporating the morpholinopyrimidine scaffold for the treatment of hepatocellular carcinoma (HCC). Among the synthesized derivatives, compound **25** emerged as the lead prototype based on superior in vitro potency and in vivo efficacy. The rational design of this series was informed by prior structure–activity relationship (SAR) studies of purine-based PI3K/mTOR inhibitors (**24**). Guided by these insights, targeted structural modifications were introduced to integrate pharmacophoric elements necessary for HDAC inhibition—specifically an appropriate linker region and a zinc-binding group (ZBG)—yielding hybrid molecules optimized for dual pathway modulation (Figure 9).

Structural modifications introduced into the PI3K-interactive region of compound **25** preserved high potency against PI3Kα; however, a ~5-fold reduction in mTOR inhibitory activity was observed relative to the parent compound. The HDAC activity profile was optimized with a six-carbon aliphatic linker, which afforded low-nanomolar potency against HDAC isoforms 1, 2, 3, 6, and 10. Compound **25** demonstrated the ability to modulate both pathways, as evidenced by increased acetylation of histone H3 and α-tubulin, along with reduced phosphorylation of Akt, across multiple cancer cell lines (PC-3, MCF-7, and HepG2). Dual-pathway modulation was further confirmed in vivo using MV4-11 and HepG2 xenograft models in NCr mice.

Importantly, compound **25** exhibited significant oral efficacy as a single agent in hepatocellular carcinoma (HCC) and metastatic breast cancer (4T1) models, coupled with favorable drug-like properties. These features supported its nomination as a promising candidate for further development, either as monotherapy or in combination regimens, with potential therapeutic applications in HCC, acute myeloid leukemia (AML), and non-Hodgkin lymphomas [56].

**Figure 10:**
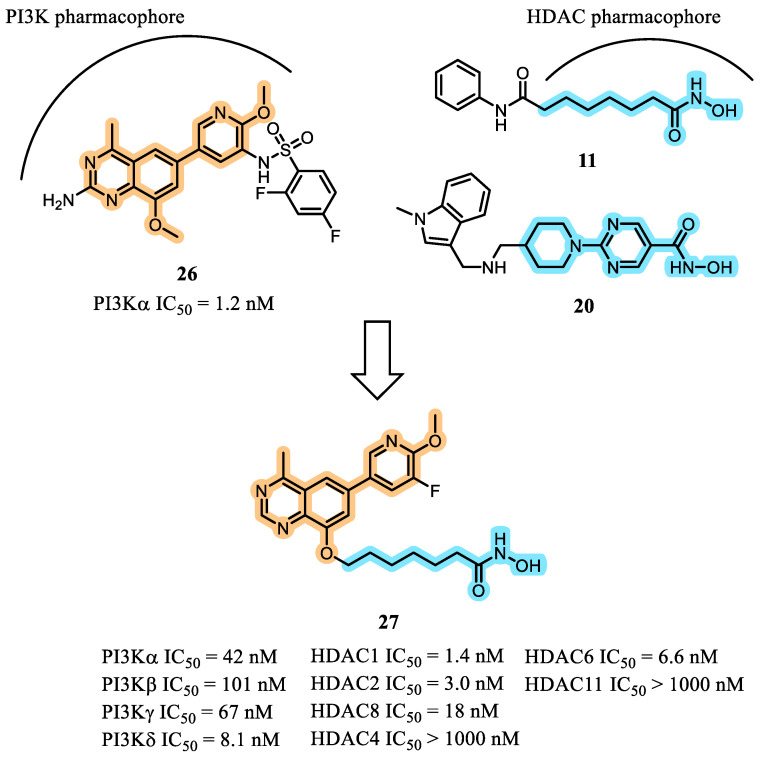
Design concept of compound **27** [57].

In 2019, Zhang et al. sought to circumvent the recurrent use of the morpholinopyrimidine scaffold by exploring a 4-methylquinazoline core for the design of dual PI3K/HDAC inhibitors [57]. Design strategy was based on the hybridization of compound **26**, previously identified as PI3K inhibitor by their group, with pharmacophoric elements characteristic of HDACis such as compounds **11** and **20** (Figure 10). Structure–activity relationship (SAR) studies were constructed by systematically modifying the linker segment attached to the hydroxamic acid zinc-binding group and by introducing substitutions on the 2-methoxypyridine ring to fine-tune PI3K activity.

Among the compounds synthesized in this series, compound **27** was selected as the lead prototype for further development due to its robust inhibitory potency against both PI3K and HDAC targets, its favorable kinase selectivity profile, and its strong antiproliferative activity across a range of cancer cell lines derived from solid tumors and hematologic malignancies (e.g., THP-1, MCF-7, U87). The compound also exhibited an acceptable preliminary safety profile. Despite less favorable pharmacokinetic characteristics—including low oral bioavailability and rapid systemic clearance, attributed primarily to metabolic instability of the hydroxamic acid group—compound **27** nevertheless demonstrated in vivo efficacy in an HCT116 human colon cancer xenograft model. Dual-pathway engagement was confirmed by increased acetylation of histone H3 and decreased phosphorylated AKT levels in tumor tissues [57].

**Figure 11:**
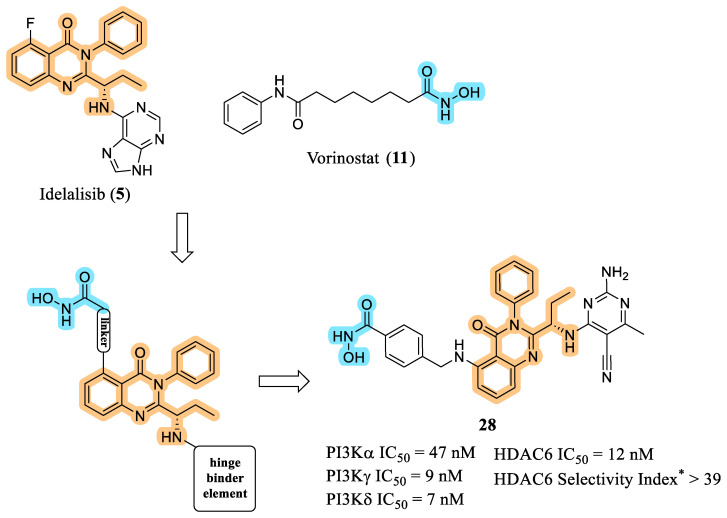
Molecular design of compound **28**. * Selectivity index relative to other HDAC isoforms [58].

In 2020, Thakur et al. reported an extensive series of dual PI3K/HDAC inhibitors featuring quinazoline or quinazolinone scaffolds as central cores, strategically integrating the essential pharmacophoric elements required for interaction with both PI3K and HDAC (Figure 11). The design strategy drew inspiration from structural features of idelalisib (**5**) and vorinostat (**11**), with systematic modifications introduced into the linker region attached to the hydroxamic acid zinc-binding group, as well as the PI3K hinge-binding fragment. The overarching aim was to generate selective and potent dual inhibitors capable of overcoming limitations associated with non-selective pan-inhibition of both HDACs and PI3Ks [58].

Extensive SAR investigations led to the identification of several potent and selective dual PI3K/HDAC inhibitors incorporating a purine moiety as the hinge-binding element, along with a linker connected to a hydroxamic acid zinc-binding group. Among these derivatives, the benzylamine-containing linker region emerged as a key structural determinant conferring preferential selectivity toward HDAC6. Despite displaying strong enzymatic inhibition profiles, this first-generation series did not exhibit satisfactory antiproliferative activity across the human tumor cell lines of the NCI-60 panel, which the authors attributed primarily to poor cellular permeability [58].

To address this limitation, a second-generation series was designed in which the purine hinge binder was replaced with a substituted aminopyrimidine fragment, with the aim of improving passive permeability and overall cell penetration. This optimization effort culminated in the identification of compound **28** as the lead prototype. Compound **28** demonstrated pronounced selectivity for HDAC6 within the HDAC family and a pan-PI3K inhibition profile, exhibiting low-nanomolar potency across both target classes. Notably, **28** showed excellent antiproliferative activity in multiple tumor cell lines and induced robust necrotic responses in AML models. In addition, the compound displayed improved metabolic stability and acceptable in vivo pharmacokinetic properties, supporting its advancement into ongoing lead optimization efforts [58].

Selective inhibition of HDAC6 in PI3K/HDAC MTDL development was the first strategy to surpass pan-HDAC inhibition associated problems. HDAC6 is quite different from the other HDAC isoenzymes, mainly due its exclusive cytoplasmic cellular location, which is associated with the modulation of non-histone proteins that are involved in cancer process (e. g., tubulin, heat shock protein 90, actin, etc.) [59,60]. Additionally, HDAC6 selective modulation may cause less toxicity since its knockout appears to not cause embryonic lethality in animal models [61].

**Figure 12:**
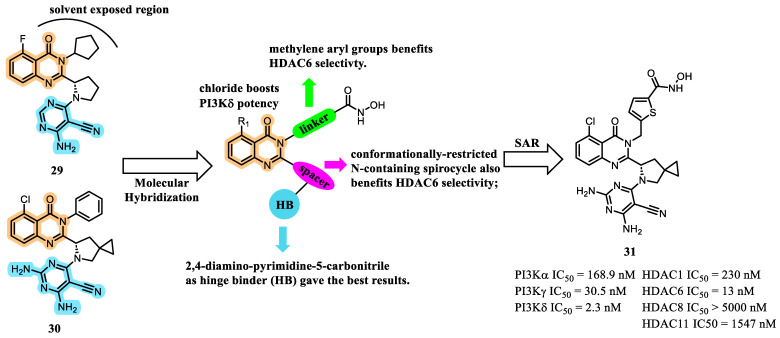
Molecular design, SAR and identification of compound **31** [59].

In a complementary approach to identify selective dual PI3K/HDAC inhibitors, Li et al., 2022, designed and synthesized a series of molecules engineered to selectively inhibit PI3Kδ and HDAC6 isoforms [62]. The design strategy was grounded in conformational analogues of structures **29** and **30**, themselves derived from the selective PI3Kδ inhibitor idelalisib (**5**), onto which HDAC-directed pharmacophores were strategically introduced (Figure 12). The decision to focus on HDAC6 selectivity stemmed from several considerations: (i) HDAC6 silencing is associated with a lower risk of lethality compared to inhibition of class I HDACs; (ii) HDAC6 regulates deacetylation of multiple non-histone substrates involved in tumor initiation, migration, and metastasis; and (iii) HDAC6 participates in immunomodulatory processes within the tumor microenvironment [33,59,63].

Similarly, targeted inhibition of PI3Kδ not only disrupts intrinsic tumor cell growth and metastatic progression, but also enhances antitumor immunity by modulating leukocyte function and tumor–immune interactions. This dual mechanism suggests a potentially synergistic therapeutic rationale for concurrent inhibition of PI3Kδ and HDAC6 isoforms [64,65].

Based on the SAR analysis, the authors observed that, with regard to PI3Kδ inhibition, a chlorine substituent at the R^1^ position conferred greater potency than fluorine. Furthermore, the 2,4-diaminopyrimidine-5-carbonitrile moiety proved to be a superior hinge-binding (HB) element compared to the purine scaffold originally employed [62]. In addition, methylene-aryl linkers and nitrogen-containing seven-membered spirocyclic spacers markedly enhanced both potency and selectivity toward HDAC6.

Within this optimized series, compound **31** demonstrated the most favorable enzymatic inhibition profile, exhibiting low-nanomolar potency across both PI3Kδ and HDAC6. The compound also displayed a strong selectivity index within the HDAC family (>18) and within the PI3K class (>13). In cell-based assays, compound **31** showed satisfactory antiproliferative activity across multiple tumor cell lines, with potencies ranging from the mid-nanomolar to low-micromolar range. Notably, in the T47D breast ductal carcinoma line, compound **31** outperformed the corresponding single-target PI3K and HDAC inhibitors by a substantial margin. The compound also exhibited acceptable cytotoxicity toward normal HUVEC cells, indicating a preliminary therapeutic window.

However, a preliminary pharmacokinetic (PK) assessment in Sprague-Dawley rats revealed unfavorable PK properties, including low oral absorption, a short half-life (0.46 h), and a clearance rate of 3.18 L/h/kg. These findings indicate the need for further structural optimization to enhance metabolic stability and systemic exposure [62].

### 4.2. Multitarget PI3K/HDAC Inhibitors Bearing o-Aminobenzamide Group

Despite the fact that most clinically approved HDAC inhibitors are hydroxamic acids, their therapeutic use is frequently limited by dose-dependent toxicities, including risks of genotoxicity and severe adverse events associated with pan-HDAC inhibition. Moreover, hydroxamate-based inhibitors exhibit suboptimal pharmacokinetic behavior due to rapid metabolic degradation and narrow therapeutic indices [66]. These limitations have driven efforts to identify alternative zinc-binding groups (ZBGs) with improved safety and pharmacological profiles. Notably, the recent approval of tucidinostat (**15**) by the CFDA has intensified interest in *o*-aminobenzamide-based HDAC inhibitors, a scaffold exemplified by entinostat (**34**), which is currently undergoing phase II/III clinical evaluation for solid tumors (NCT02115282) and hematologic malignancies (NCT03179930), and which received CFDA approval in 2024 for the treatment of advanced or metastatic breast cancer (NCT03538171).

**Figure 13:**
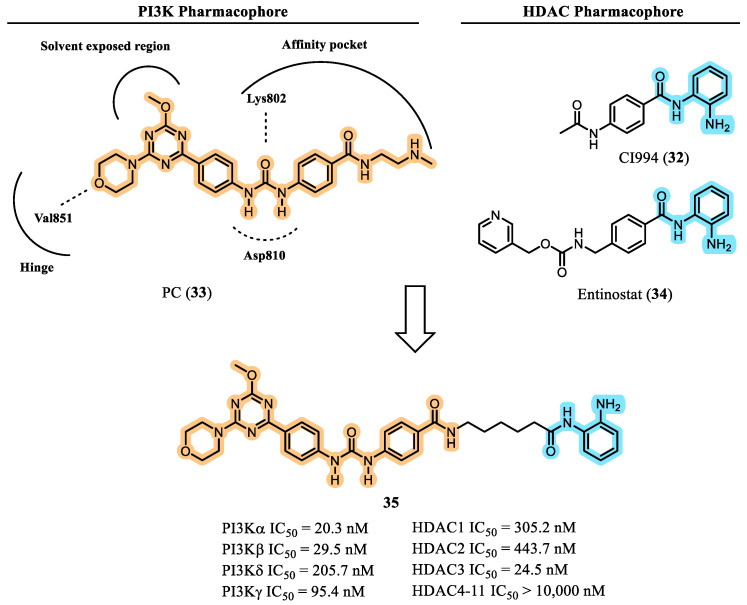
Concept design and identification of PI3K/HDAC multitarget inhibitor **35** [64].

Seeking alternatives to hydroxamate ZBGs for dual PI3K/HDAC modulation, Deng et al. (2023) designed a novel series of multitarget inhibitors incorporating a benzamide group as the zinc-binding pharmacophore [67]. Their strategy was guided by structural analyses of the PI3K inhibitor PC (**33**), which contains a morpholinotriazine core, and by benzamide-based HDAC inhibitors such as CI-994 (**32**) and entinostat (**34**). Through a rational molecular hybridization approach, the authors generated hybrid molecules that preserved the essential pharmacophoric features required for activity at each target: (i) a morpholine moiety to anchor within the PI3K hinge region, (ii) a urea fragment positioned to establish hydrogen-bond interactions at the PI3K affinity site, and (iii) a benzamide ZBG capable of forming the characteristic bidentate coordination with the catalytic zinc ion in the HDAC active site via its carbonyl oxygen and aniline nitrogen (Figure 13) [67].

Following extensive SAR evaluation, compound **35** emerged as the lead molecule, exhibiting potent multitarget activity against class I HDAC isoforms (HDAC1 IC_50_ = 305.2 nM, HDAC2 IC_50_ = 443.7 nM, and HDAC3 IC_50_ = 24.5 nM) as well as broad PI3K inhibition (PI3Kα IC_50_ = 20.3 nM, PI3Kβ IC_50_ = 29.5 nM, PI3Kδ IC_50_ = 205.7 nM, and PI3Kγ IC_50_ = 95.4 nM). In cell-based antiproliferative assays, compound **35** demonstrated low-micromolar activity across multiple tumor cell lines, including MM1.S (multiple myeloma), Jeko-1 (mantle cell lymphoma), HL-60 (acute promyelocytic leukemia), and MDA-MB-231 (triple-negative breast cancer). Notably, **35** was more potent than the reference HDAC inhibitor CI-994 (**29**) and the PI3K inhibitor PC (**28**), and displayed no detectable cytotoxicity in normal human liver HL-7702 cells.

Dual-target engagement by compound **35** was confirmed through its ability to downregulate phosphorylated AKT while increasing acetylated histone H3 levels. The absence of tubulin acetylation indicated selective inhibition of class I HDACs rather than HDAC6. Moreover, compound **35** induced apoptosis in Jeko-1 cells—a response not observed with either reference inhibitor **32** or **33**—highlighting the functional importance of simultaneous HDAC and PI3K inhibition [67].

**Figure 14:**
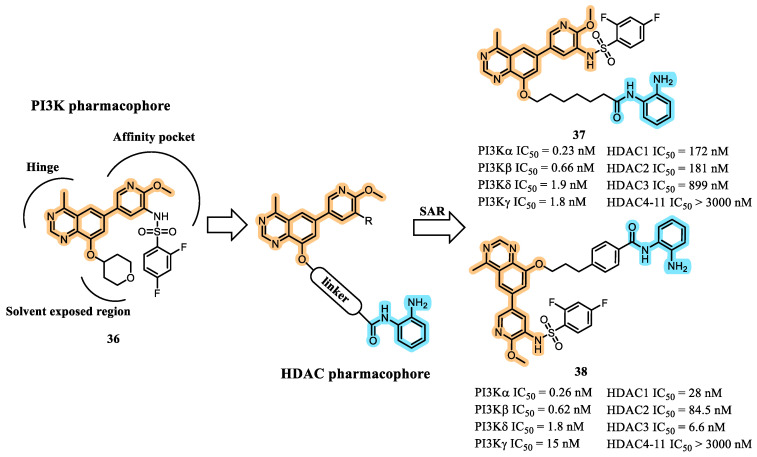
Molecular design of benzamide multitarget PI3K/HDAC inhibitors **37** and **38** [65].

To identify new dual PI3K/HDAC inhibitors for the treatment of acute myeloid leukemia (AML), Zhang et al., 2024, designed a series of 4-methylquinazoline derivatives incorporating a benzamide zinc-binding group (ZBG) [68]. Their design strategy involved analyzing the binding mode of compound **36**, a PI3K inhibitor featuring the 4-methylquinazoline scaffold. Structural modeling revealed that, in addition to establishing canonical interactions at the hinge region and affinity pocket, the C-8 position of the quinazoline ring was oriented toward the solvent-exposed region, providing an optimal site for structural extension (Figure 14) [68].

From the SAR analyses, compounds **37** and **38** emerged as the most promising dual inhibitors, exhibiting the strongest simultaneous enzymatic inhibition of PI3K and class I HDAC isoforms, along with the most potent antiproliferative activities across a broad panel of hematologic cancer cell lines. These included acute myeloid leukemia (AML) cell lines (MV4-11, NB4, HL-60), non-Hodgkin lymphoma (NHL) cell lines (DOHH2, Ramos, Raji), and mantle cell lymphoma (MCL) cells (Mino). In most of these models—particularly in MV4-11—compounds **37** and **38** demonstrated markedly superior antiproliferative activity relative to the reference HDAC inhibitors tucidinostat (15) and vorinostat (11).

Western blot and flow cytometry analyses confirmed effective dual-pathway engagement for both compounds, evidenced by reduced phosphorylated AKT levels and increased acetylated histone H3 in MV4-11 cells. These pharmacodynamic signatures validated the intended simultaneous PI3K and HDAC inhibition.

In vivo pharmacokinetic studies in ICR mice revealed that both **37** and **38** possessed a mean half-life of approximately 2.35 h. Although oral bioavailability remained low, the overall systemic exposure (AUC) following intravenous and oral administration was superior to that of comparable hydroxamate-based analogues, indicating improved metabolic stability.

Importantly, compounds **37** and **38** exhibited potent, dose-dependent antitumor activity in MV4-11 xenograft models in nude mice, further supporting their potential as viable candidates for continued preclinical development [68].

## 5. Summary and Outlook

The emergence of multitarget-directed ligands (MTDLs) has reshaped therapeutic strategies for multifactorial diseases, particularly in oncology, where conventional single-target approaches frequently fail to overcome the complexity and adaptability of tumor biology. This paradigm shift underscores the growing necessity for drug design methodologies capable of simultaneously modulating multiple signaling pathways implicated in cancer progression, thereby enhancing therapeutic efficacy while potentially mitigating toxicity and resistance mechanisms. Within this context, the dual modulation of epigenetic regulators and kinase-driven signaling cascades—most notably through concurrent inhibition of HDACs and PI3Ks—has gained increasing prominence as a rational and promising strategy for anticancer drug development.

The discovery of fimepinostat (CUDC-907, **21**), the first-in-class PI3K/HDAC multitarget inhibitor to enter phase I/II clinical trials, catalyzed extensive research efforts aimed at producing next-generation dual modulators with diverse selectivity profiles and refined pharmacological properties. Although many of these compounds have demonstrated compelling biological activity in vitro and in vivo, challenges related to pharmacokinetics, pharmacodynamics, and safety—particularly those associated with hydroxamic acid zinc-binding groups—remain major obstacles to clinical translation.

Hydroxamates, despite their high-potency, are frequently limited by rapid metabolic clearance, poor oral bioavailability, and concerns regarding genotoxicity. The major factor that leads to mutagenicity caused by hydroxamic acids is a Lossen rearrangement, which converts a hydroxamate to its isocyanate derivative. This species is unstable and can react with nucleophiles such as DNA nucleophilic groups [66].

These limitations have driven a clear trend toward the exploration of alternative scaffolds capable of providing more selective, stable, and safer interactions with the HDAC catalytic zinc ion. The recent regulatory approvals of tucidinostat (**15**) and entinostat (**34**) by the CFDA have strengthened interest in *ortho*-aminobenzamide–based ZBGs as viable substitutes for hydroxamates, particularly considering their improved pharmacological tolerability and therapeutic potential in both solid and hematologic malignancies.

Collectively, the progress summarized in this review demonstrates that dual PI3K/HDAC inhibition remains a highly promising therapeutic avenue. Continued optimization of scaffold design, ZBG selection, and target selectivity holds substantial potential to yield next-generation multitarget agents capable of addressing unmet clinical needs in oncology.

## Figures and Tables

**Table 1:** Enzyme inhibition data for HDAC and PI3K for fimepinostat (**21**) and its prototypes **19** and **20** [46,47,48].

	IC_50_ (nM)										
	HDACs										
Compound	**1**	**2**	**3**	**8**	**4**	**5**	**7**	**9**	**6**	**10**	**11**
Quisinostat (**20**)	0.11	0.33	4.86	4.26	0.64	3.69	119	32.1	76.8	0.46	0.37
Fimepinostat (**21**)	1.7	5.0	1.8	191	409	674	426	554	27	2.8	5.4
	PI3Ks										
Compound	**α**			**β**		**δ**			**γ**		
Pictilisib (**19**)	8			31		4			55		
Fimepinostat (**21**)	19			54		39			311		

**Table 2:** Active clinical trials with multitarget HDAC/PI3K inhibitors 21 and 22.

Inhibitor	Realizador	Indication	Phase	NCT Number	Start Date
Fimepinostat (**21**)	University of California, Los Angeles	Cushing disease	II	NCT05971758	16 January 2025
	University of California, San Francisco	Diffuse Intrinsic Pontine Glioma, Recurrent Medulloblastoma, or Recurrent High-Grade Glioma	I	NCT03893487	7 August 2019
	Dana-Farber Cancer Institute	Solid Tumors, Brain Tumors or Lynphoma	I	NCT02909777	October 2016
Ifupinostat (**22**)	BeBetter Med Inc.	DLBCL R/R	III	NCT06792253	6 January 2025
	BeBetter Med Inc.	DLBCL R/R	II	NCT06074107	15 June 2020
	BeBetter Med Inc.	DLBCL R/R	I	NCT06164327	1 December 2023

## Data Availability

No new data were created or analyzed in this study.
